# Exploring Dental Fusion in Primary Dentition: A Pediatric Dental Perspective

**DOI:** 10.7759/cureus.68469

**Published:** 2024-09-02

**Authors:** Mridula Goswami, Sakshi Lohia

**Affiliations:** 1 Pediatric and Preventive Dentistry, Maulana Azad Institute of Dental Sciences, New Delhi, IND; 2 Pediatric Dentistry, Maulana Azad Institute of Dental Sciences, New Delhi, IND

**Keywords:** young child, pediatric preventive dentistry, oral health care, dental anomalies, dental fusion

## Abstract

Developmental anomalies of dentition includes abnormalities in teeth size, shape, structure, and number. Dental fusion is one such condition and is one of the most important and frequent developmental dental anomalies that can aﬀect children’s oral health. This condition can present various clinical challenges, including aesthetic concerns, occlusal disturbances, and potential impacts on the development and eruption of permanent teeth. The etiology of dental fusion is multifactorial, involving genetic, environmental, and possibly mechanical factors during tooth development. Diagnosis is done based on clinical examination and radiographic imaging to differentiate fusion from other similar anomalies such as gemination and concrescence. The present case report describes dental fusion in primary teeth seen in two young pediatric patients. These case reports aim to provide an overview of the prevalence, diagnosis, and management strategies for fusion in primary teeth, emphasizing the importance of a Pediatric Dentist in optimizing outcomes for affected children.

## Introduction

A dental anomaly is an irregularity or deviation from the normal characteristics of the dentition. It encompasses a wide range of variations that can affect the size, shape, structure, number, and positioning of teeth. These anomalies may be congenital, arising from developmental disturbances during tooth formation, or acquired due to environmental factors, trauma, or pathological conditions affecting the oral cavity [1]. One intriguing occurrence within this realm is dental fusion, a phenomenon where two adjacent tooth germs unite during their development, resulting in the formation of a single, larger tooth structure. Such anomaly can be complete or incomplete depending on the time of developmental stage at which the associated tooth buds are [2]. Complete fusion happens if two dental buds come into contact before the calcification phase, manifesting clinically as a single, large crown. The fusion may be restricted to the roots only if it occurs during the advanced stage of morph-differentiation, which would mean that the fused teeth would have distinct pulp chambers and root canals [3]. Fusion in primary dentition is mostly observed unilaterally [4]. Dental fusion in primary teeth is a rare developmental anomaly that presents unique challenges for dental professionals, especially bilateral fusion of lateral incisor and canine. When two tooth germs fuse, they create a distinctive clinical and radiographic appearance that requires careful examination and consideration for appropriate management. The crown, pulp chamber, or root unites the teeth, and this fusion can be complete (total) or incomplete (partial), depending on the stage of differentiation, when it happened. Fused teeth might contain separate pulp canals or share a common pulp canal. Fusion can happen between two normal teeth or between a normal tooth and a supernumerary tooth. The crown appears large and lacks any distinct groove, forming a complete fusion with a pulp chamber that appears normal. The pulp chamber may seem bifid or split in an incomplete fusion, and the separating groove may be seen. The union of dentin is the primary criterion, and the degree of fusion relies on the stage of tooth development that has happened at the time of fusion [5]. The fused teeth are often asymptomatic and are diagnosed during routine dental examination. Problems related to abnormalities in primary teeth include malocclusion, an unesthetic appearance of the involved teeth, a higher risk of caries, and difficult eruption of the succeeding teeth [6].

The etiology of dental fusion is multifactorial and can be influenced by various factors. Maternal factors such as nutrition, exposure to toxins, or infections may impact tooth development, potentially increasing the risk of dental fusion. Trauma or mechanical forces during the formative stages of tooth development can lead to fusion. Injuries to the developing oral structures, such as pressure or impact, may cause adjacent tooth germs to fuse. Certain genetic syndromes or developmental disorders are associated with dental anomalies. Syndromes like Ellis-van Creveld syndrome and Down syndrome, among others, may include dental fusion as part of their clinical manifestations.

The worldwide incidence of fused teeth ranges from 0.14% to 5.0% [7]. Primary lower incisors are most commonly affected, with a prevalence of fused teeth in primary dentition of 0.5% to 1% compared to 0.01% to 0.2% in permanent dentition. Dental fusion occurs more often in the mandible's anterior region than the maxillary arch, and it can have bilateral or unilateral presence [8]. Fusion can happen between teeth in the same dentition, between mixed dentition or even between teeth that are normal and supernumerary [9]. Literature suggests that there is no significant difference between males and females whereas studies have suggested a racial indicating a higher incidence among the Japanese and Chinese population compared to the Caucasian population [10]. Compared to European and European-derived people, Asian and Asian-derived populations have a higher occurrence of fused teeth [11]. A study conducted on 1062 people (15-30 years old) in Odisha, India regarding dental abnormalities related to the maxillary lateral incisor revealed a 0.18% frequency of fusion [12].

The present cases describe the intricate merging of primary tooth germs during development, leading to a singular structure with fused clinical crowns, their management and follow-ups in two pediatric patients.

## Case presentation

Case 1

**Figure 1:**
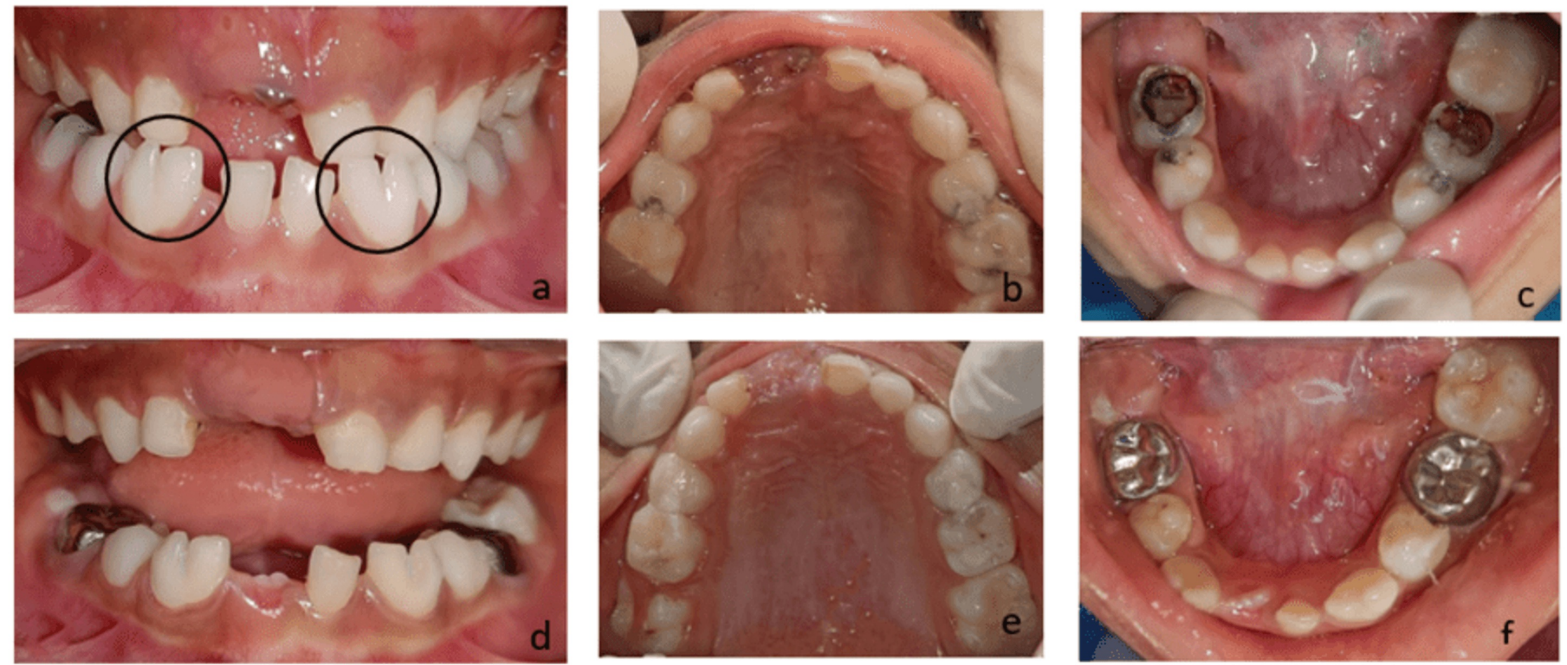
Case 1 clinical images A – Pre-operative frontal view showing fusion of crowns with respect to 72,73 and 82,83, B – Maxillary occlusal view, C – Mandibular occlusal view, D – Pit and fissure sealants with respect to 72,73 and 82,83, E – Restoration with respect to 54,64,65, F – Pulpectomy followed by stainless steel crowns with respect to 75 and 85, restoration with respect to 74,84 at six-month follow-up

**Figure 2:**
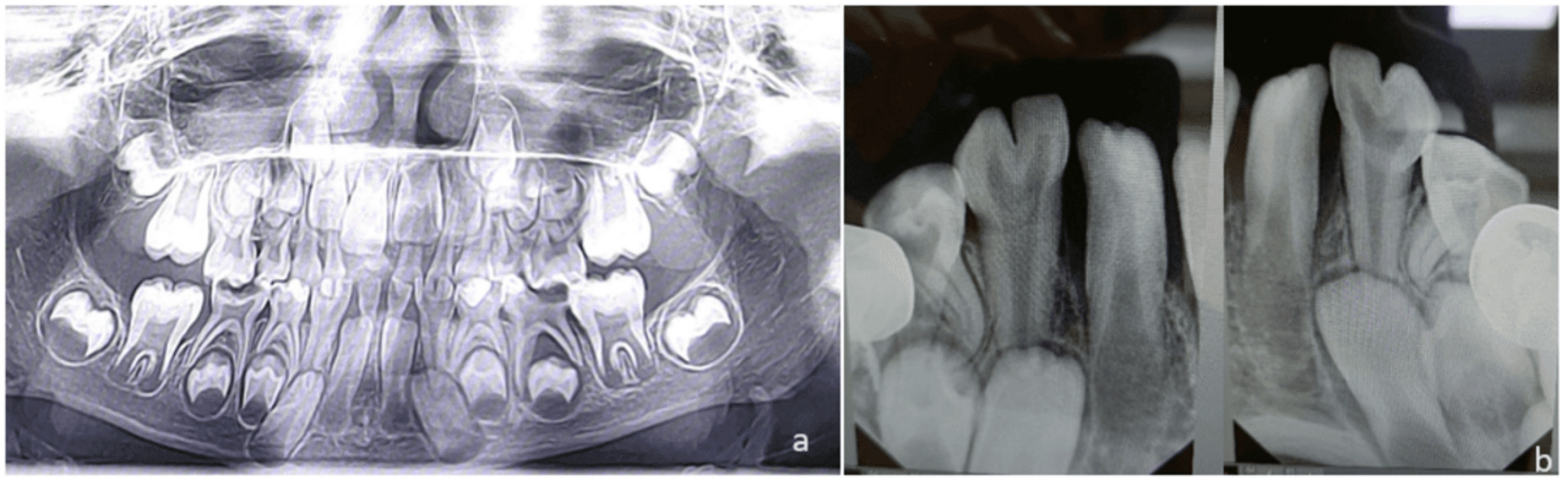
Case 1 radiographic images A - Orthopantomogram revealing fusion with respect to 72,73 and 82,83; Agenesis of tooth buds 32 and 42. B - Radiovisualgraph revealing type I fusion with respect to 72,72 and 82,83 with absence of tooth bud of lateral incisor bilaterally

A five-year-old female patient accompanied by her mother reported to the outpatient department, Pediatric and Preventive Dentistry, Maulana Azad Institute of Dental Sciences (MAIDS), New Delhi with a chief complaint of pain and sensitivity in the lower tooth region for two months. The child's medical history did not provide any relevant information, and there were no previous incidents of dental injury. Patient has one younger sister, who does not have any similar or other dental anomaly on examination. The behaviour of the patient on initial visit was ‘Negative’ (rating 2) as per the Frankl behaviour rating scale. This child had mixed dentition with exfoliative mobility present with respect to (wrt) lower central incisors and root stump wrt 51. Clinical examination revealed grossly carious 75, 85; carious 74,84 and fusion of clinical crowns present between 72,73 and 82,83 (Figure 1). Orthopantomogram (OPG) and radiovisiography (RVG) radiograph wrt fused teeth revealed the absence of tooth buds wrt 32 and 42 (Figure 2A, 2B). Planned treatment included root canal therapies in primary teeth with irreversible pulpitis, followed by restoration with stainless steel crowns and preventive therapy. Patient’s behaviour management was done using non-pharmacological behaviour management techniques such as ‘Tell-Show-Do’, ‘Audio-Visual distraction’, ‘Modelling’ and a positive dental attitude was observed during subsequent appointment. Extraction of the retained root stump wrt 51 was done under local anaesthesia. Pit and fissure sealants (Clinpro; 3M, Saint Paul, MN, USA) application were done in the developmental groove demarcating crowns of 72,73 and 82,83. Patient is kept on regular follow-ups for maintenance phase of the treatment. Patient education was done regarding maintenance of oral hygiene care at home.

Case 2

**Figure 3:**
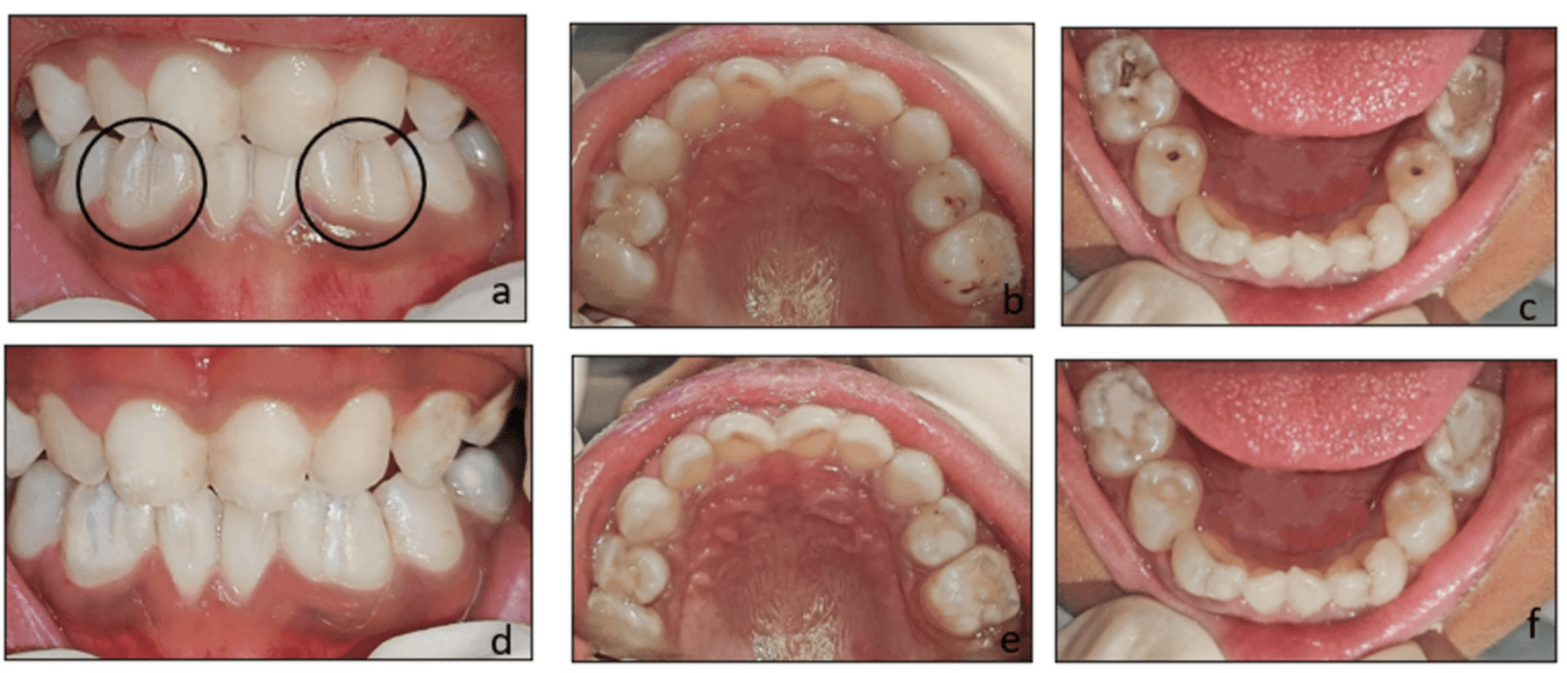
Case 2 clinical images A – Pre-operative frontal view showing fusion of 72,73 and 82,83, B – Maxillary occlusal view, C – Mandibular occlusal view, D – Pit and fissure sealants with respect to 72,73 and 82,83, E – Restoration with respect to 64,65, F – Restoration with respect to 74,75 and 84,85 at six-month follow-up

**Figure 4:**
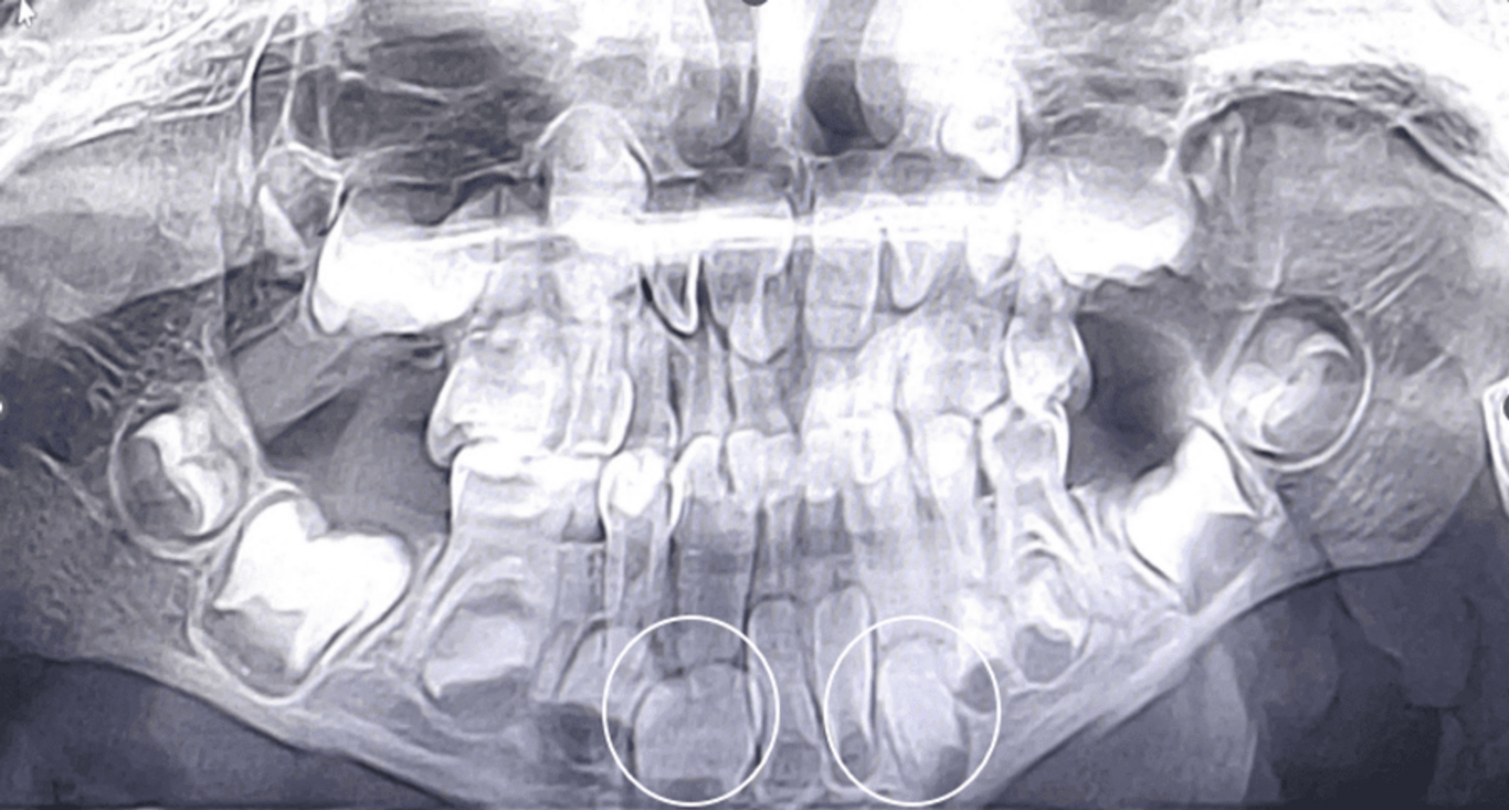
Case 2 radiographic image Orthopantomogram revealing fusion with respect to 72,73 and 82,83; Agenesis of tooth buds 32 and 42

A four-year-old female patient reported with a chief complaint of pain while chewing food from the lower right tooth region for one week. Patient had delayed milestones as per average height and weight of the age group. Medical history of the patient was non-significant. The behaviour of the patient was fearful and ‘Negative’ (Rating 1) as per the Frankl behaviour rating scale. Behaviour management was done in the subsequent visits by gradually desensitizing the patient to dental environment using ‘Tell Show Play Do’ and with positive reinforcement in the form of praise and toys. Clinical examination revealed grossly carious 75,85; carious 74,84 and fusion present between 72,73 and 82,83 (Figure 3). Presence of fused crowns was confirmed via intraoral periapical radiographs (IOPAs) and OPG, which further revealed the absence of tooth buds of permanent lateral incisors (Figure 4). On enquiring about any problem in relation to fused teeth, the patient had history of sensitivity in the same region. The first few visits of the patient were directed towards behaviour management using non-pharmacological approaches and simpler procedures such as oral prophylaxis and counselling. On later visits when patient showed ‘Positive’ behaviour (rating 3) as per the Frankl behaviour rating scale, indirect pulp capping of 75 and 85 was done followed by restoration with glass ionomer cement (Espe Gi filling cement; 3M). Pit and fissure sealants (Clinpro; 3M) were applied for the fused teeth and primary molars. Patient was kept on regular follow-ups and re-application of sealants was done at six-months follow-up.

In both cases, parent and patient counselling was done regarding the dental anomaly and preventive strategies such as maintenance of oral hygiene and fluoride usage; regular dental checkups were done and advised long-term follow-up.

## Discussion

The etiology of dental fusion, the union of two adjacent tooth germs during development, is multifactorial and can be influenced by various genetic, environmental, and developmental factors (Table 1).

**Table 1:** Etiology  of Dental Fusion

S.No	Etiology of Dental Fusion
1.	Genetic
2.	Environmental: Nutritional deficiency, Exposure to toxins, Maternal health during pregnancy
3.	Trauma
4.	Hereditary or familial patterns

Understanding the etiological factors, prevalence, clinical implications, and potential complications associated with dental fusion in primary teeth is crucial for dental practitioners. The role of Pediatric Dentists is very important as they are the ones to recognize such anomalies at younger ages. Management done in the early stages results in better long-term prognosis and the patient and parents can both can be counselled about the condition and possible outcomes in permanent dentition. A study reported complications mostly observed primary fused teeth (PFT) were at higher risk of caries and tooth aplasia fusion. Another observation was the majority of patients with PFT (85%) also exhibited other developmental dental anomalies such as peg-shaped incisors, talon cusps, and ectopic and delayed eruption of the permanent teeth [2]. Primary fused teeth are mostly observed unilaterally [4]. Bilateral tooth fusion occurrence is rare with a 0.02% prevalence rate in deciduous dentition [13]. In the present cases, fusion of primary teeth was observed bilaterally in relation to the mandibular lateral incisor and canine. This anomaly not only poses aesthetic concerns but may also impact the eruption patterns, occlusion, and overall oral health of affected individuals. Dental fusion can be associated with a genetic tendency and agenesis of its permanent successor. Both the presented cases revealed absence of lateral incisor tooth buds bilaterally. Comprehensive clinical and radiographic evaluations are important to detect developmental dental anomalies earlier in young children and this has always been challenging. The management becomes challenging as behaviour management of such a young age group of children is difficult. We used non-pharmacological behaviour management techniques in managing both the patients by gradually desensitizing them to dental treatment and instilling a positive attitude. The abnormal alignment of fused teeth may affect the overall occlusion, leading to issues such as crowding or misalignment. Orthodontic intervention may be required to address these issues and achieve proper alignment. Fused teeth may have irregular surfaces and grooves that can be difficult to clean properly. This can increase the risk of dental caries and periodontal disease. Children with fused teeth should be monitored closely for signs of decay, and preventive measures such as fluoride treatment and dental sealants are recommended as a preventive measure. The most adequate dental intervention is from a multidisciplinary approach involving a Pediatric Dentist, Orthodontist, and Prosthodontist considering the child’s condition, expectation, and degree of cooperation with dental treatment. Regular follow-up visits are crucial for the fused tooth cases despite the absence of signs and symptoms. Furthermore, the incidence of fusion teeth occurring in a similar region and similar teeth with positive family history indicates supporting the evidence of the shared genetic control of dental developmental disturbances.

## Conclusions

Dental fusion results from the physical union of two tooth buds during their development. Radiographs help differentiate between fusion and other anomalies like gemination i.e. incomplete splitting of a single tooth bud, concrescence (teeth joined by cementum), and macrodontia (enlarged teeth). The fusion of two adjacent teeth during development can lead to a larger, irregularly shaped tooth, often resulting in noticeable aesthetic concerns, particularly when it occurs in the front teeth. This can impact an individual's self-esteem and social interactions, especially among children and adolescents. Functionally, dental fusion can cause malocclusion or misalignment of teeth, affecting chewing and speaking abilities, and potentially leading to crowding or spacing issues that may require orthodontic treatment. Additionally, the irregular morphology of fused teeth poses challenges for oral hygiene, as plaque tends to accumulate in areas that are difficult to clean, increasing the risk of dental caries and periodontal disease.

Future studies on the prevalence and patterns of dental fusion across different populations can provide insights into the environmental and genetic factors influencing this condition. This will help in identifying and developing preventive strategies. Education and awareness about dental fusion help in early detection and better compliance with dental care routines. Ongoing dental monitoring is vital to prevent potential complications, ensuring healthier long-term outcomes.

## References

[1] Rajendran R (2009). Shafer’s Textbook of Oral Pathology.

[2] Açıkel H, İbiş S, Şen Tunç E (2018). Primary fused teeth and findings in permanent dentition. Med Princ Pract.

[3] Pereira AJ, Fidel RA, Fidel SR (2000). Maxillary lateral incisor with two root canals: fusion, gemination or dens invaginatus?. Braz Dent J.

[4] Cheng RB, Chen X, Liu SJ, Pan L, Wu XG (2003). [An epidemiological survey on fusion of deciduous teeth of 4286 kindergarten children in Shenyang city]. Shanghai Kou Qiang Yi Xue.

[5] Goh V, Tse OD (2020). Management of bilateral mandibular fused teeth. Cureus.

[6] Goswami M, Bhardwaj S, Grewal N (2020). Prevalence of shape-related developmental dental anomalies in India: a retrospective study. Int J Clin Pediatr Dent.

[7] Babaji P, Prasanth MA, Gowda AR, Ajith S, D'Souza H, Ashok KP (2012). Triple teeth: report of an unusual case. Case Rep Dent.

[8] Bernardi S, Bianchi S, Bernardi G, Tchorz JP, Attin T, Hellwig E, Karygianni L (2020). Clinical management of fusion in primary mandibular incisors: a systematic literature review. Acta Odontol Scand.

[9] Zengin AZ, Celenk P, Gunduz K, Canger M (2014). Primary double teeth and their effect on permanent successors. Eur J Paediatr Dent.

[10] Hattab FN (2014). Double talon cusps on supernumerary tooth fused to maxillary central incisor: review of literature and report of case. J Clin Exp Dent.

[11] Wu CW, Lin YT, Lin YT (2010). Double primary teeth in children under 17 years old and their correlation with permanent successors. Chang Gung Med J.

[12] Shashirekha G, Jena A (2013). Prevalence and incidence of gemination and fusion in maxillary lateral incisors in Odisha population and related case report. J Clin Diagn Res.

[13] Duncan WK, Helpin ML (1987). Bilateral fusion and gemination: a literature analysis and case report. Oral Surg Oral Med Oral Pathol.

